# Correlation between Sun Protection Factor and Antioxidant Activity, Phenol and Flavonoid Contents of some Medicinal Plants 

**Published:** 2014

**Authors:** Mohammad Ali Ebrahimzadeh, Reza Enayatifard, Masoumeh Khalili, Mahdieh Ghaffarloo, Majid Saeedi, Jamshid Yazdani Charati

**Affiliations:** a *Pharmaceutical Sciences Research Center, School of Pharmacy, Mazandaran University of Medical Sciences, Sari, Iran*; b *Department of Pharmaceutics, School of Pharmacy, Mazandaran University of Medical Sciences, Sari, Iran. *; c *Department of Biostatistics, Faculty of Health, Mazandaran University of Medical Sciences, Sari, Iran. *

**Keywords:** Crataegus pentagyna, Feijoa sellowiana, Sambucus ebulus, Corn silk, Sun Protection factor

## Abstract

Long exposure of UV radiation increases risk of skin diseases such as cancer and photoallergic reactions. UV-B (280-320 nm) radiation is mainly responsible for inducing the skin problems. Skin protection is a suitable method against ultraviolet radiation-induced damage. Various synthetic agents have been used as photo protective but because of their potential toxicity in humans, they have limited usage. Natural substances have been recently considered as potential sunscreen resources due to their absorption in the UV region and their antioxidant activity. In the present study, the UV protective effects of 20 extracts from four common medicinal plants were evaluated. Their phenol and flavonoid contents and antioxidant activities were determined and correlation between SPF and these contents were evaluated. SPFs were between 0.102 and 24.470. The highest value was reached with ultrasonic extract of *Crataegus pentagyna *(SPF = 24.47) followed by methanolic extract of *Feijoa sellowiana *(SPF = 1.30). Good correlation was found between SPF and phenolic contents (Correlation Coefficient = 0.55 and p = 0.01) but no correlations were found between SPF and flavonoid contents or antioxidant activity. These extracts can be used alone or as additives in other sun screen formulations to enhance their SPF.

## Introduction

Chronic exposure of human skin to solar ultraviolet (UV) radiation may cause several skin damages. These damages include sunburn, skin cancer and oxidative stress as well as photoaging depending on the amount and form of the UV radiation and on the type of the individual exposed (1). The ultraviolet region of the electromagnetic spectrum can be divided into three regions: UVA, from 320 to 400 nm; UVB, from 290 to 320 nm and UVC, from 200 to 290 nm (2). UVC radiation is filtered by the atmosphere before reaching the earth. UVB radiation is not completely filtered out by the ozone layer and is responsible for the damages due to sunburn. UVA radiation reaches the deeper layers of the epidermis and dermis and provokes the premature aging of the skin (3). 

Sunscreens are now incorporated into products such as moisturizers, creams, lotions and other hair and skin preparations. The regular use of these products may help reduce the chance of the harmful effects of ultraviolet radiation. A sunscreen’s ability to block UV-B is more important for the prevention of negative effects of sun exposure (4). However, it is necessary that a very efficient sunscreen substance is used in the cosmetic formulation. The efficacy of a sunscreen is usually expressed by the sun protection factor (SPF), which is defined as the UV energy required for producing a minimal erythema dose (MED) on protected skin, divided by the UV energy required producing a MED on unprotected skin. MED is defined as the lowest time interval or dosage of UV light irradiation sufficient for producing a minimal, perceptible erythema on unprotected skin (3). The higher the SPF, the more protection a sunscreen offers against sunburn.

There is now an increasing body of evidence that the use of sunscreen is not entirely safe for sunscreen protection (5, 6). Natural products are therefore important sources for research in new active compounds. This offers the possibility of discovering new biological mechanisms, to obtain new active molecules, and to study their structure function relationships in order to develop more active drugs and to avoid unwanted side effects. Furthermore, if the natural substance sources are common and widely occurring, it is possible to produce a high quantity at a low price (7-9). Natural substances have been recently considered as potential sunscreen resources because of their absorption in the UV region (10) and their antioxidant activity (11). Green tea polyphenols, *Aloe barbadensis *extract, aromatic compounds isolated from *lichens*, glycosides of *aesculin *and *Murrayakoenigii *leaf essential oil are examples of natural substances evaluated for their sunscreen specifications (12-16). There is strong evidence that DNA-damaging ultraviolet (UV) light induces the accumulation of UV light-absorbing flavonoids and other phenolics in dermal tissue of the plant body. This suggests physiological function, yet speculative in light protection in plants and of course in human (17). There has been an increasing interest in the use of antioxidants in sunscreens to provide supplemental photo protective action activity. Antioxidants from natural sources may provide new possibilities for the treatment and prevention of UV-mediated diseases (18, 19).

The Iranian flora is rich in medicinal plants with a high potential for providing these antioxidants. Among them, *Sambucus ebulus *(traditional name: Palem)*, Zea maize, Feijoa sellowiana *and *Crataegus pentagyna *(traditional name: Siah-Valik)are four medicinal plants which their high antioxidant activity has been reported recently (20-23). In spite of many reports of sun screen activity from medicinal plants, there are no data available about correlation between phenol/flavonoid contents or antioxidant activity and their SPF. The goals of this research are to evaluate: 1) SPF of 20 extracts from four medicinal plants; 2) their phenol and flavonoid contents; 3) their antioxidant activities and 4) correlation between SPF and these contents.

## Experimental

*Plant material and preparation of freeze-dried extract*

Corn Silk (dried cut stigmata of *Zea maysL*,) and *S. ebulus, F. sellowiana *and C*. pentagyna *fruits were obtained in summer of 2011 from Sari, Iran. The sample was authenticated by Dr. Bahman Eslami (plant systematic specialist)and the voucher specimen (No. 275-278) has been deposited in the Sari School of Pharmacy herbarium. Plant material was dried under dark condition at room temperature for 2 weeks. The dry material was milled, obtaining 2-3 mm particles and then extracted by methanol (HPLC grade, Merk, Germany) and water (Deionized, Millipore, France), separately for 24 h at room temperature. The extracts were then separated from the sample residues by filtration through Whatman No. 1 filter paper and repeated three times. The resulting extracts were concentrated over a rotary vacuum at 35-40°C until a crude solid extract was obtained which was then freeze-dried (MPS-55 Freeze-drier, Cperon, Korea) for complete solvents removal (For yields see Table 1).

**Table 1:** Sun protection factor, total phenol contents, total flavonoid contents and antioxidant activities of *S. ebulus, Zea maize, F. sellowiana *and *C. pentagyna*

**Plant name/Extraction method**	**Extraction yield (%)**	**Phenol contents (GAE /g of extract)**	**Flavonoid contents (QE /g of extract)**	**Antioxidant activity (IC**_50_ **)**	**SPF ** **(at 2 mg /mL)**
S. ebulus					
Aqueous extract	20.5	355.17	21.31	95.56	0.715
Methanolic extract	34.2	585.51	83.75	82.73	0.202
Polyphenol fraction	4.9	1374.48	52.28	86.30	0.428
Soxhlet extract	41	166.50	31.25	88.76	0.165
Ultrasonic extract	21.8	90.00	27.91	142.24	0.224
Zea maize					
Aqueous extract	29.9	212.75	19.78	77.67	0.269
Methanolic extract	20.4	264.31	96.25	73.21	0.377
Polyphenol fraction	3	875.86	103.68	91.36	0.417
Soxhlet extract	40.28	45.50	19.58	179.67	0.335
Ultrasonic extract	24	38.00	14.33	481.15	0.166
F. sellowiana					
Aqueous extract	16.5	541.72	24.62	95.23	0.559
Methanolic extract	10	375.24	110.31	92.41	1.298
Polyphenol fraction	2.9	717.24	60.71	93.87	0.479
Soxhlet extract	16.7	274.50	22.08	427.91	0.187
Ultrasonic extract	4.86	216.50	22.5	697.89	0.222
C. pentagyna					
Aqueous extract	24.1	249.13	12.75	92.77	0.102
Methanolic extract	24	186.72	85.31	50.44	0.123
Polyphenol fraction	3.1	246.89	70.87	83.92	0.417
Soxhlet extract	28.3	137.00	37.08	17.48	0.152
Ultrasonic extract	16.26	136.50	56.66	22.81	24.47

GAE: Gallic acid equivalent; QE:Quercetin equivalent.

*Preparation of polyphenol fraction*

Samples were extracted according to our recently published paper (23). The extractions were performed twice at 20 °C in a shaking incubator. Extracting time was 30 min and extracting solvent was 100 mL of methanol/acetone/water (3.5/3.5/3) containing 1% formic acid. All extracts were collected and evaporated under vacuum at 35-40 °C to remove methanol and acetone. Lipophilic pigments were then eliminated from the aqueous phase by extraction with petroleum ether. The aqueous phase was collected and further extracted three times by ethyl acetate. Organic phases were collected and concentrated over a rotary vacuum until a crude solid extract was obtained, which was then freeze-dried for complete solvent removal and used as polyphenol (PP) fraction (For yields see Table 1).

*Ultrasonically assisted extraction*

Samples were extracted with methanol in an ultrasonic bath over 1 h through indirect sonication at a frequency of 100 kHz and a temperature of 25 ± 3°C for 1 h to yield ultrasonic extracts. The extracts were then separated from the samples residue by filtration. The resulting extracts were concentrated in a rotary evaporator until crude solid extracts were obtained which were freeze-dried for complete solvent removal and used as ultrasonic (US) extracts (23) (For yields see Table 1).

*Soxhlet assisted extraction*

The powders of samples were extracted exhaustively in a Soxhlet extractor with methanol. The extracts were then concentrated in a rotary evaporator until the solvent was completely removed. The methanol extract was kept in a well-closed container in refrigerator until use (24).

*Determination of total phenolic and flavonoid contents*

Total phenolic content was measured colorimetrically using the Folin-Ciocalteu reagent (25). Results were expressed as gallic acid equivalents (GAE). The standard curve was prepared by 25, 50, 100, 200, 250, 400 and 500 μg mL^-1^ solutions of gallic acid in methanol: water (50:50, v/v). Each sample was analyzed in triplicate (standard curve was Y=0.0058X, R^2^ = 0.989).Total flavonoids were estimated using aluminum chloride method (25). Total flavonoid contents were calculated as quercetin from a calibration curve.The calibration curve was prepared by preparing quercetin solutions at concentrations 15.62 to 250 μg ml^-1^ in methanol (standard curve was Y=0.0064X – 0.0076, R^2^ = 0.999). The experiment was repeated for three times.

*DPPH radical-scavenging activity*

1,1-diphenyl-2-picryl hydrazyl radical (DPPH) was used for determination of free radical-scavenging activity of the extracts (26). One mL of methanolic solution of DPPH (100 μM) was added to 1 mL of different concentrations (25-800 μg/mL) of each extract. After 15 min in dark at room temperature, the absorbance was recorded at 517 nm. 

*Calculation of in-vitro SPF*

SPF by definition is determined *in-vivo *as the increase in exposure time required to induce erythema, *i.e. *SPF 4 means four times longer to induce erythema. The most common *in-vitro *technique involves measuring the spectral transmittance at UV wavelengths from 280 nm to 400 nm (27). Screening of sun protection activity was measured by determination of the *in-vitro *SPF. The extracts were dissolved in methanol. Scanning spectra of the samples in solution were obtained by running from 337.5 to 292.5 nm (at 5 nm intervals). The SPF determination model used in this study was based on the following equation proposed by Gharavi *et al*. (28).

SPF=∑337.5 E(λ)ε(λ)∑337.5 EλελT (λ)

 Here, T (λ) is the measured sunscreen transmittance at λ; E (λ) is the spectral irradiance of terrestrial sunlight at λ; and ε (λ) is the erythemal action spectrum at λ. The values of the E (λ) and ε (λ) were calculated according to the report by Gharavi *et al. *(28). T (λ) was measured three times for each extract and the mean was used for calculation of SPF. At least, five different concentrations of each extract were used for obtaining the standard curve and calculating SPF in 2 mg/mL of solution.

*Statistical analysis*

Data were compared with Normal Density by Kolmogorov-Simonov test and their associations were analyzed by Spearman correlation coefficient. Data were analyzed statistically by SPSS 20.The experiment was repeated for three times.

## Results

The SPF is a quantitative measurement of the effectiveness of a sunscreen formulation. To be effective in preventing sunburn and other skin damages, a sunscreen product should have a wide range of absorbance between 290 and 400 nm. Evaluation of the efficiency of a sunscreen has been assessed for a long time through *in-vivo *test which is performed on human volunteers. The *in-vitro *SPF is useful for screening test during the product development, as a supplement of the *in-vivo *SPS measure (3).

Most spectrophotometric techniques for transmittance measurements rely on preparing samples with a uniform and known thickness so that the optical path length through the sample is standardized. Many samples are dissolved in special solvents and placed into 10 mm path length cuvettes. The cuvettes for UV spectrophotometry are usually made from quartz which is transparent to UV wavelengths (29). In the solvent method, different concentrations of test products in methanol were prepared. Each sample’s transmittance was measured to evaluate the SPF value Sunscreens containing a wide variety of chemicals that have specific absorbance in some parts of the UV spectrum. Plant extracts, due to containing a wide range of natural compounds, usually cover full range of UV wavelengths. One approach to protecting the body from the harmful effects of UV irradiation is to use active photoprotectives.

In recent years, naturally occurring compounds have gained considerable attention as protective agents (30, 31). There is at least one literature review about the photoprotective effects of some naturally occurring herbal such as polyphenols (30). Phenolics are believed to be capable of acting in redox-sensitive signaling cascades to inhibit DNA damage. The phenolics may be beneficial in preventing UV-induced oxygen free radical generation and lipid peroxidation, *i.e. *events involved in pathological states such as photo aging and skin cancer (17). Antioxidant activity is important in UV protection (32). It is reported that high SPF values of *D. moldavica*and *V. tricolor *(24.79 and 25.69 respectively) may be due to their high phenolic contents (32). High concentration of flavonoids such as rutin in plants may be used to prevent UV-induced oxygen free radical generation, too (33). On the other hand, UV is highly genotoxic and many studies have unanimously mentioned that the mutations generated in critical growth control genes by the UV component of sunlight, are the first stage in skin cancer development (32, 34). To prevent such DNA damage induced by UV that penetrates into skin cells, the anti mutagens having the potentials against UV is likely to be considered. To cut down UV radiation, one possible way is using anti-mutagens which are active towards UV-induced mutation. A number of herbs and their components have been found to have the anti-mutagenic potentials with their proposed mechanisms of action (32, 35-38). Although UV radiation has some benefits, its negative effect in human health is much more. Skin cancer is one of the serious results. UV is a strong physical mutagen. Its penetration into skin cells is likely to cause gene mutation and this is believed to be the first stage in skin cancer development (39).

**Figure 1:**
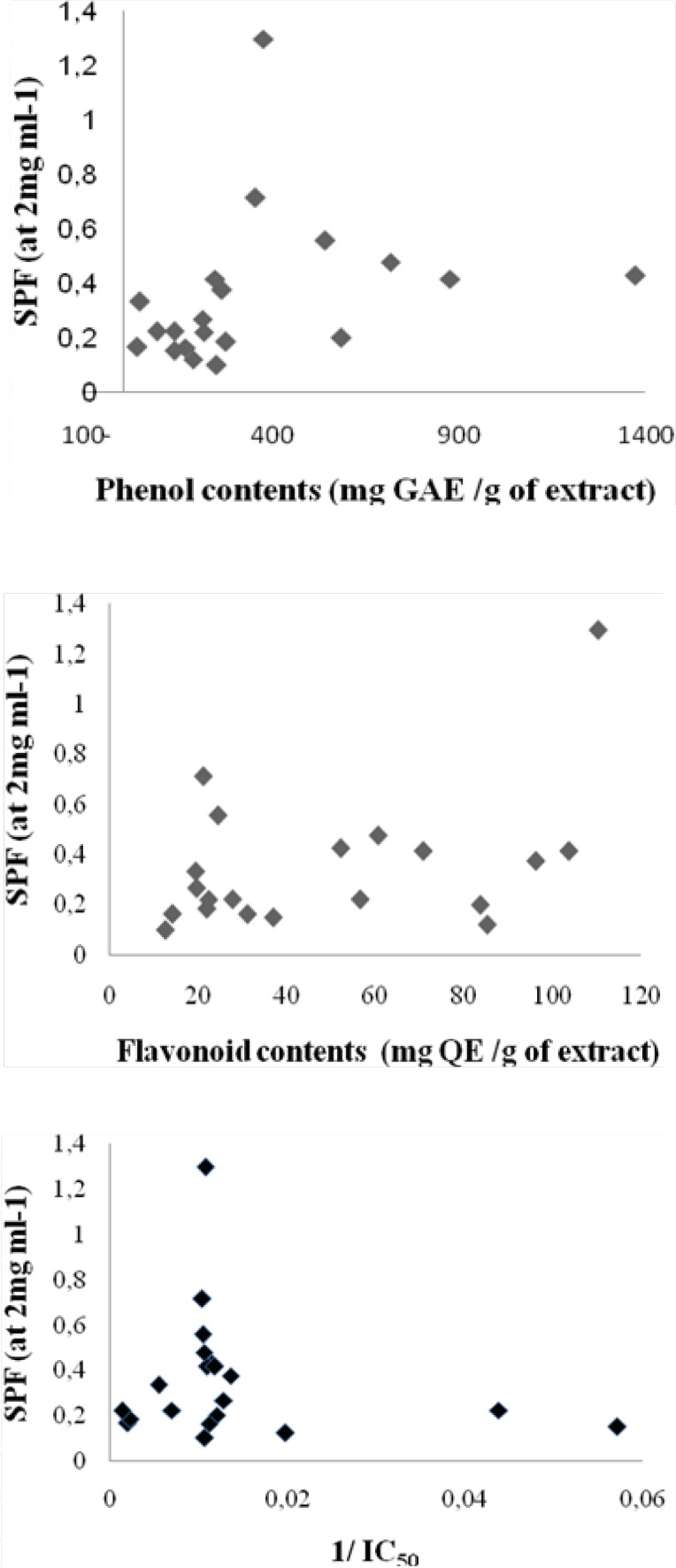
Correlation between SPF and phenolic contents (upper), flavonoid contents (middle) and antioxidant activity (by DPPH test)(lower).

In this research, 20 extracts from four known antioxidant medicinal plants were evaluated for their SPF by UV spectrophotometry applying Gharavi mathematical equation (28). Table 1 shows the SPFs of extracts at 2 mg/mL concentrations in methanol. The protection factors were between 0.102 and 24.470. The highest value was reached using ultrasonic extract of *Crataegus pentagyna *(SPF = 24.47) followed by methanolic extract of *Feijoa sellowiana *(SPF = 1.30). Sun protective activities of the rest of the plants were low representing by their sun protection being lower than 1. Figure 1 shows correlations between SPF and phenolic contents, flavonoid contents and antioxidant activity (by DPPH test). Good correlation was found between SPF and phenolic contents (Correlation Coefficient = 0.55 and p = 0.01) but no correlations were found between SPF and flavonoid contents (Correlation Coefficient = 0.30 and p = 0.19) or antioxidant activity (Correlation Coefficient = 0.14 and p = 0.56) in tested extracts.

## Conclusion

In this study, it was proved that ultrasonic extract of *Crataegus pentagyna *has high sun protection effect. This extract can be used as additives in other sun screen formulations to enhance their SPF. Good correlation was found between SPF and phenolic contents but no correlations were found between SPF and flavonoid contents or antioxidant activity.According to high SPF value of this extract, its anti-mutagenic activity should be considered.
